# Aging, Alzheimer's disease, and stroke: A 25-year longitudinal analysis of U.S. mortality trends

**DOI:** 10.1177/13872877251380689

**Published:** 2025-09-24

**Authors:** Muhammad Faizan Ali, Husnain Ahmad, Mohamed Fawzi Hemida, Muhammad Faizan Tahir, Talha Qadeer, Sana Rasheed, Zarwa Rashid, Mohammad Hamza Bin Abdul Malik, Ashraf Ahmed, Alexander Morden, Muhammad Abdullah Naveed, Himaja Dutt Chigurupati, Sivaram Neppala

**Affiliations:** 1 Department of Cardiology, Jinnah Postgraduate Medical Center, Karachi, Pakistan; 2 Department of Cardiology, Shalamar Medical and Dental College, Lahore, Pakistan; 3 Department of Cardiology, Faculty of Medicine, Alexandria University, Alexandria, Egypt; 4 Department of Cardiology, Jinnah Sindh Medical University, Karachi, Pakistan; 5 Department of Cardiology, King Edward Medical University, Lahore, Pakistan; 6 Department of Cardiology, Nassau University Medical Center, New York City, USA; 7 Department of Cardiology, Department of Internal Medicine, Yale University School of Medicine, Bridgeport Hospital, Bridgport, CT, USA; 8 Department of Cardiology, Dow Medical College, Karachi, Pakistan; 9 Department of Cardiology, East Carolina University, Greenville, NC, USA; 10 Department of Cardiology, UT Health Science Center, San Antonio, TX, USA

**Keywords:** age-adjusted mortality, Alzheimer’s disease, geographic variations, racial disparities, rural versus urban health, stroke, stroke-related mortality

## Abstract

**Background:**

Alzheimer's disease (AD) and stroke, both of which are age-related conditions, exhibit common risk factors such as hypertension, smoking, diabetes, and the *APOE* ε4 genotype, which frequently coexist in older adults.

**Objective:**

This study investigates disparities in stroke-related mortality among AD patients, categorized by sex, race/ethnicity, and geographic region.

**Methods:**

The age-adjusted mortality rates (AAMRs) per 100,000 for adults 65 and older were sourced from the CDC WONDER database using ICD codes for AD (G30) and stroke (I60–I61, I63–I64, I69). Joinpoint regression estimated the Annual Percent Change (APC) and Average Annual Percent Change (AAPC), with statistical significance at p < 0.0001.

**Results:**

From 1999 to 2023, there were 154,323 deaths related to strokes in AD patients, primarily occurring in nursing homes (53.4%). The AAMRs decreased from 19.4 in 1999 to 10.5 in 2023 (APC: −2.6%, p < 0.01). Women exhibited higher AAMRs than men (15.9 versus 12.6), although men experienced a steeper decline (AAPC: −2.7%). Non-Hispanic (NH) Blacks reported the highest AAMR at 16.9, while NH Whites demonstrated the most considerable decline (AAPC: −2.7%, p < 0.01). AAMRs varied considerably, ranging from 27 in Mississippi to 6 in New York, with the Northeast region reflecting the most significant decline (AAPC: −3.5%). Furthermore, rural areas displayed higher AAMRs than urban regions (19.4 versus 14.3), although both populations exhibited declining trends.

**Conclusions:**

Stroke mortality in AD patients has decreased but remains unevenly distributed, especially among women, NH Black individuals, rural communities, and Western U.S. residents. Targeted interventions are essential.

## Introduction

Stroke and Alzheimer's disease (AD) are among the leading causes of mortality and disability in the United States (U.S). In 2020, strokes were responsible for approximately 1 in every 19 deaths, with one person experiencing a stroke every 40 s and a related death occurring every 3.5 min.^1^ Simultaneously, around 6.2 million Americans aged 65 and above are living with AD, a figure expected to nearly double by 2050.^2^ Notably, deaths due to AD increased by 145% between 2000 and 2019, underscoring its escalating impact on public health.^3^

Substantial evidence highlights a significant connection between stroke and neurodegenerative processes. Stroke and AD share multiple modifiable risk factors, such as hypertension, diabetes mellitus, and smoking.^4^ When these conditions coexist, they tend to accelerate cognitive decline and lead to poorer functional outcomes. Research shows that nearly 30% of stroke survivors develop dementia within a year.^5^ Moreover, patients with both stroke and AD experience faster deterioration and higher mortality rates compared to those affected by either condition alone.^6,7^ Vascular injury is also implicated in the accumulation of amyloid plaques and tau proteins, further linking the stroke with AD progression.^8^

Despite known associations, large-scale longitudinal analyses examining the joint impact of stroke and AD on mortality trends remain limited. Previous research has predominantly examined these conditions separately, limiting understanding of their combined burden.^6,9^ This study addresses this gap by analyzing 25 years of U.S. mortality data, aiming to support the development of integrated public health strategies to reduce avoidable deaths linked to aging, stroke, and dementia.

## Methods

### Study setting and population

In this retrospective study, we utilized publicly available death certificate data retrieved from the CDC WONDER (Centers for Disease Control and Prevention Wide-Ranging Online Data for Epidemiologic Research) database.^10^ We analyzed death certificate data from 1999 to 2023 for stroke-related mortality in individuals with AD aged 65 years or older, using the following International Statistical Classification of Diseases and Related Health Problems-10th Revision (ICD-10) codes: Stroke (I60.0, I60.1, I60.2, I60.3, I60.4, I60.5, I60.6, I60.7, I60.8, I60.9, I61.0, I61.1, I61.2, I61.3, I61.4, I61.5, I61.6, I61.8, I61.9, I63.0, I63.1, I63.2, I63.3, I63.4, I63.5, I63.6, I63.8, I63.9, I64, I69.0, I69.1, I69.3, I69.4) and Alzheimer's Disease (G30.0, G30.1, G30.2, G30.8, G30.9). Consistent with prior research, the ICD codes utilized in our study to identify stroke and AD mirror those employed in preceding investigations.^11,12^ The Multiple Cause-of-Death Public Use records were analyzed, and deaths related to stroke and AD were identified. This was based on the presence of stroke and AD listed anywhere on the death certificate, whether as a contributing factor or an underlying cause of death.

To uphold the quality of our reporting, we adhered to the Strengthening the Reporting of Observational Studies in Epidemiology (STROBE) guidelines in this observational study.^13^ Additionally, our study complied with the American Heart Association (AHA) Guidelines on Racial and Ethnic Reporting, thereby ensuring a standardized and accurate portrayal of racial and ethnic groups within our analysis.^14,15^ This study utilized publicly available, de-identified mortality data from the CDC WONDER database,^10^ which does not require institutional review board (IRB) approval or special administrative permissions. All data use complied with the CDC's terms of access and ethical standards for public data analysis.

### Data abstraction

State, year, region, population size, demographics, site of death, and urban-rural classification were among the abstracted data. The demographics included the place of death, race/ethnicity, sex, and age. The data were further organized based on the place of death. In the course of this investigation, all death locations observed in the database were categorized into three groups: Hospital or Nursing Home (Inpatient, Outpatient or Emergency Room, Dead on Arrival, Unknown Status, Long-Term Care Facility), Home or Hospice (Decedent's residence, Hospice facility), and Other (Other, Place of death unknown). The following categories of race and ethnicity were used: non-Hispanic (NH) White, NH Black or African American, Hispanic or Latino, NH American Indian or Alaskan Native, NH Asian or Pacific Islander. This information, derived from death certificates, aligns with prior WONDER database analyses.^16^ The National Center for Health Statistics Urban-Rural Classification Scheme was used to assess the population by urban (large metropolitan area [population > 1 million] and rural (population < 50,000) counties per the 2013 US census classification.^17^ The U.S. Census Bureau's division of regions into the Northeast, Midwest, South, and West was used to classify the regions.

### Selection criteria

This study included:

– U.S. adults aged 65 and above.
– The state registries reported deaths between 1999 and 2023, with issued death certificates to the CDC-WONDER database.
– Deaths due to AD and Stroke.

This study excluded:

– Deaths not reported to state registries.

### Statistical analysis

Crude and age-adjusted mortality rates (AAMRs) were calculated per 100,000 populations from 1999 to 2023 by year, sex, race/ethnicity, state, and urban-rural status with 95% confidence intervals (CIs). This provided a better understanding and allowed us to analyze national trends in stroke- and AD-related mortality in the US. The indirect method of estimating age-standardized mortality rates used the US Census 2000 as the standard population.^18^ Crude mortality rates were computed by dividing the overall number of stroke-related deaths among AD patients by the corresponding U.S. population for that year. To evaluate national yearly trends in stroke-related mortality in AD patients, the Joinpoint Regression Program (Joinpoint V 5.2.0.0, National Cancer Institute) was used to determine the Annual Percent Change (APC) and Average Annual Percent Change (AAPC) with a 95% CI in AAMR.^19^ This allows identification of significant changes in AAMR over time by fitting log-linear regression models where temporal variation occurred. APCs were considered to increase or decrease if the slope describing the shift in mortality significantly differed from zero using 2-tailed t-testing. A value of p < 0.05 was considered statistically significant. All analyses were conducted using complete case data available from the CDC WONDER database^10^

Suppressed or unavailable data were excluded from analysis, and no data imputation methods were applied.

## Results

**Figure 1.**
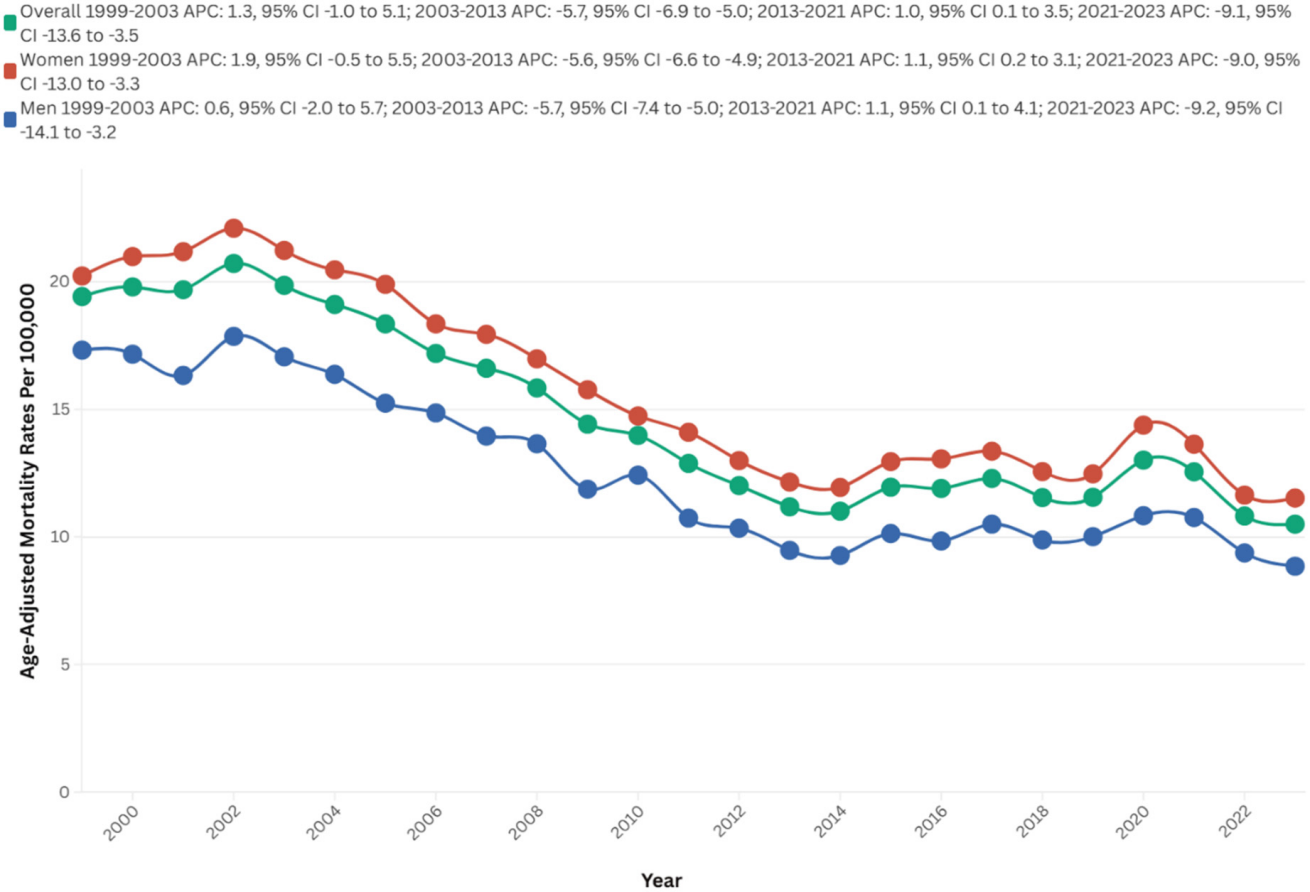
Overall and sex-stratified AAMRs per 100,000 of stroke among old (65+) AD patients in the United States (1999–2023).

Between 1999 and 2023, strokes among AD patients resulted in 154,323 deaths in the U.S. The AAMR initially exhibited no clinically significant change, increasing from 19.4 in 1999 to 19.9 in 2003 (APC = 1.3; 95% CI: −0.9 to 5.1, p = 0.23). Subsequently, there was a consecutive decrease to 11.2 in 2013 (APC = −5.7; 95% CI: −6.9 to 5.0, p < 0.01), followed by a slight increase to 12.6 in 2021 (APC = 1.0; 95% CI: 0.1 to 3.5, p = 0.03). Finally, the AAMR experienced a significant decline to 10.5 in 2023 (APC = −9.1; 95% CI: −13.6 to −3.5, p < 0.01) (Figure 1, Supplemental Tables 1 and 2).

Mortality data, when stratified by place of death, revealed significant disparities. Nursing homes and long-term care facilities accounted for 53.5% of deaths, followed by residences at 20.1%, medical facilities at 16.5%, and hospice facilities at 9.9% (Supplemental Table 3).

### Demographics

#### Gender

Mortality trends stratified by gender revealed significant disparities, with women experiencing a higher overall number of deaths compared to men (105,745 versus 48,578). Women also exhibited a higher overall AAMR over the study period (15.9 versus 12.6 per 100,000). Additionally, AAPC indicated a steeper decrease in mortality rates among men (AAPC: −2.7, 95% CI: −3.2 to −2.3, p < 0.01) compared to women (AAPC: −2.5, 95% CI: −2.8 to −2.1, p < 0.01).

A comprehensive analysis elucidated significant temporal variations in AAMR between the male and female populations. The AAMR exhibited no clinical significance within the female cohort from 1999 to 2003 (APC = 1.9; 95% CI: −0.5 to 5.5, p = 0.11). This was followed by a marked decline to 12.2 in 2013 (APC = −5.6; 95% CI: −6.6 to −4.9, p < 0.01), a subsequent slight increase to 13.6 in 2021 (APC = 1.1; 95% CI: 0.2 to 3.1, p = 0.02), and ultimately a decrease to 11.5 in 2023 (APC = −9.0; 95% CI: −12.3 to −3.3, p = 0.001)

In males, the AAMR initially exhibited no clinical significance from 1999 to 2003 (APC = 0.6; 95% CI: −2.0 to 5.7, p = 0.6). This was succeeded by a substantial decline to 9.5 in 2013 (APC = −5.7; 95% CI: −7.4 to −5.0, p < 0.01). Subsequently, there was an increase to 10.8 in 2021 (APC = 1.1; 95% CI: 0.1 to 4.1, p = 0.03) and ultimately decrease to 8.9 in 2023 (APC = −9.2; 95% CI: −14.1 to −3.2, p = 0.0004) (Figure 1, Supplemental Tables 1, 2, and 4)

#### Race

**Figure 2.**
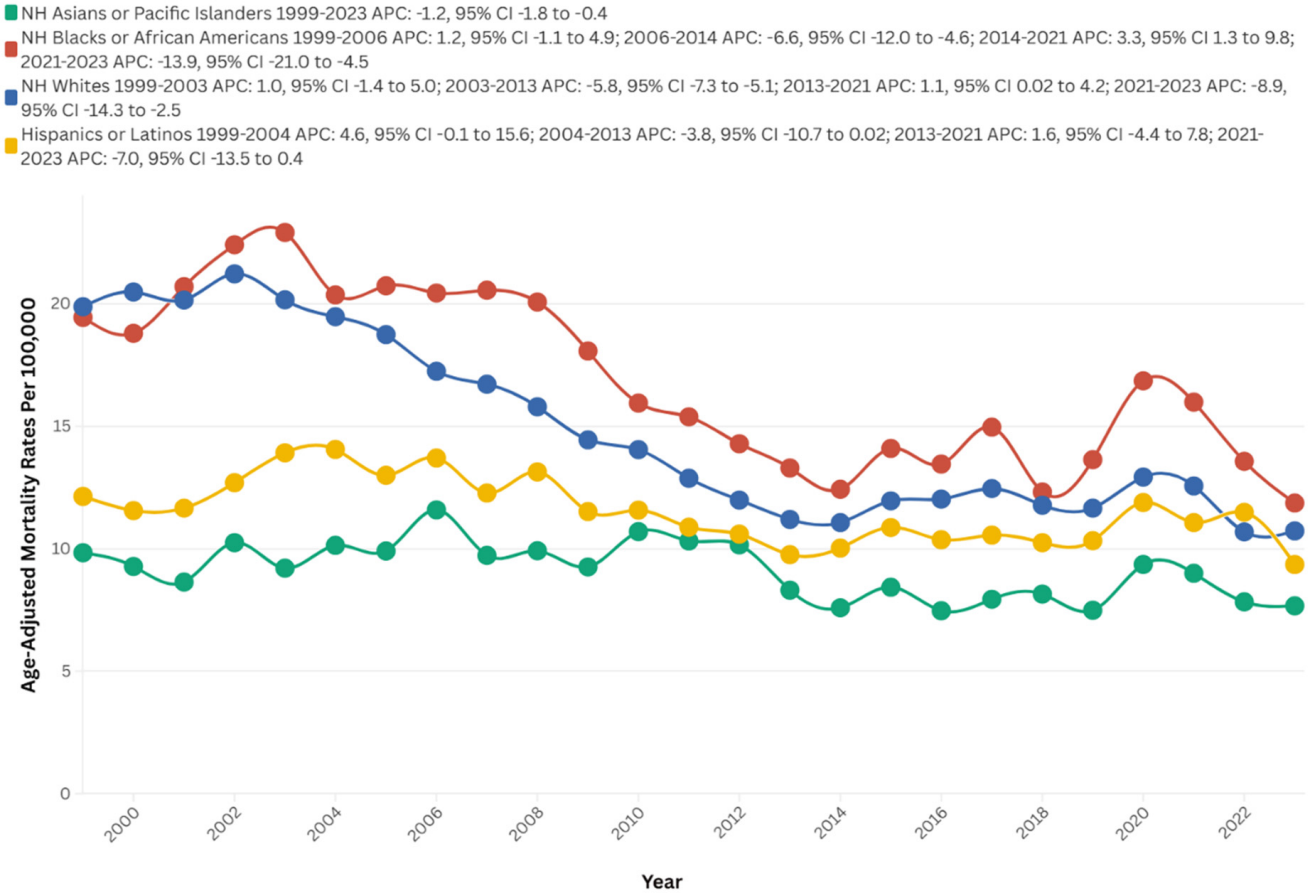
Race-stratified AAMRs per 100,000 of stroke among old (65+) AD patients in the United States (1999–2023).

Between 1999 and 2023, NH Whites exhibited the highest number of deaths, totaling 128,830. In contrast, NH Black or African American individuals displayed the highest AAMR at 16.9, followed by NH Whites at 14.9, Hispanic or Latino individuals at 11.5, and NH Asian or Pacific Islander individuals at 9.1. The most significant reductions in mortality rates were observed during the period from 2021 to 2023 for NH Blacks or African Americans (APC: −13.9), NH Whites (APC: −8.9), and Hispanic or Latino individuals (APC: −7.0). For Black individuals, the most substantial increase occurred between 2014 and 2021 (APC: 3.3), whereas for NH Whites, the steepest rise was observed from 2013 to 2021 (APC: 1.1), and for Hispanic or Latino individuals, the increase was noted between 1999 and 2004 (APC: 4.6), respectively. NH Whites experienced the most pronounced decrease in mortality over time (AAPC: −2.7, 95% CI: −3.2 to −2.3; p < 0.01), followed by NH Blacks (AAPC: −2.2; 95% CI: −2.8 to −1.5; p < 0.01) and NH Asian or Pacific Islander individuals (AAPC: −1.2; 95% CI: −1.8 to −0.4; p < 0.01) (Figure 2, Supplemental Table 1, 2, and 5).

### Geographic location

#### Census

From 1999 to 2023, the majority of deaths occurred in the Southern region (58,255), followed by the West (39,617), the Midwest, and the Northeast (19,971). Conversely, the highest AAMR recorded across all regions was in the West at 17.9, followed by the South at 15.9, the Midwest at 15.0, and the Northeast at 9.2.

The West showed no clinical significance from 1999 to 2003 (APC = 2.0; 95% CI: −0.4 to 5.9, p = 0.1), followed by a notable decrease to 14.1 in 2014 (APC = −4.1; 95% CI: −5.2 to −3.5, p = 0.001). There was no clinical significance again until 2021 (AAPC = 0.3; 95% CI: −0.9 to 3.2, p = 0.43), ultimately decreasing to 13.2 in 2023 (APC = −7.0; 95% CI: −10.9 to −2.1, p < 0.01).

**Figure 3.**
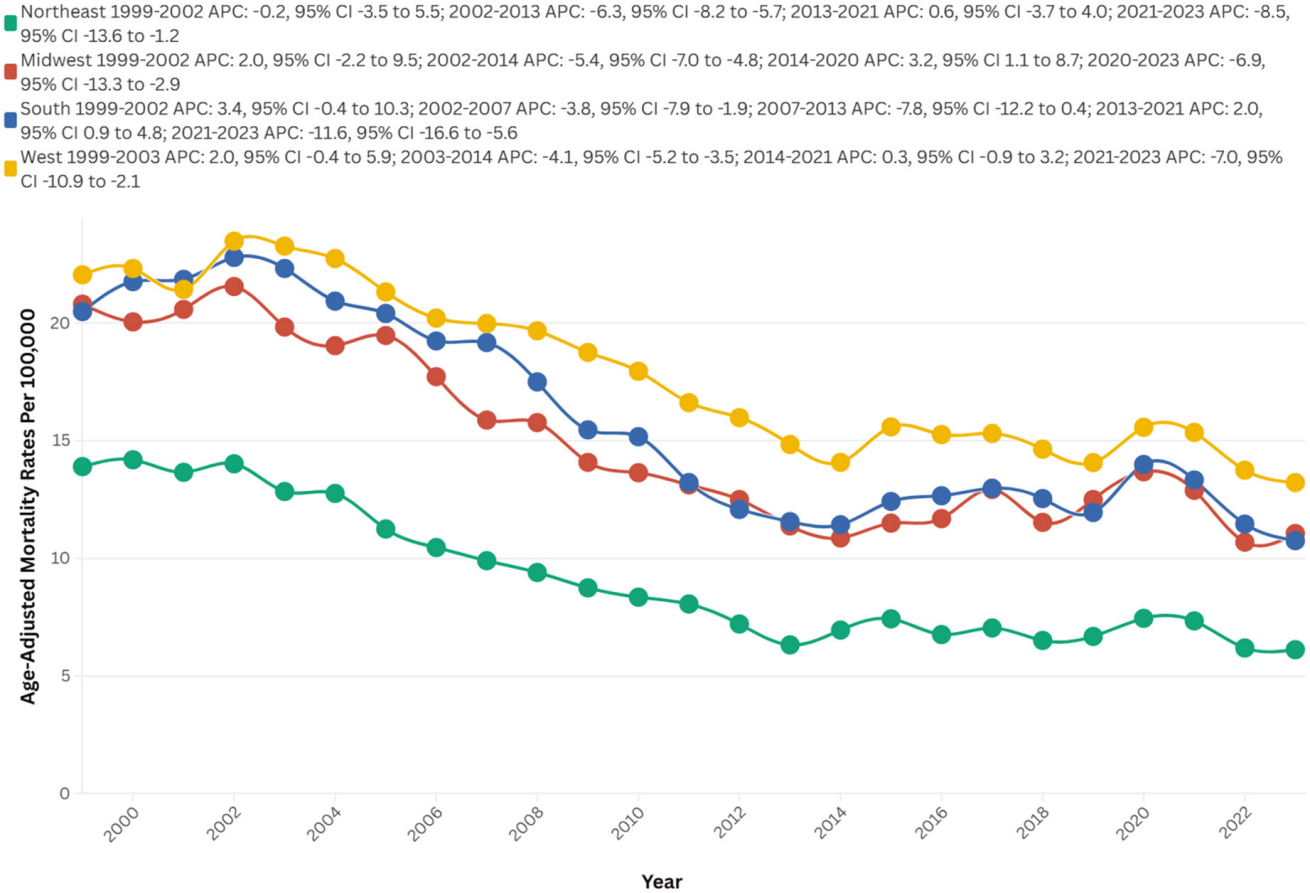
AAMRs per 100,000, by U.S. Census Regions, of stroke among old (65+) AD patients in the United States (1999–2023).

The Northeast initially showed no significant changes from 1999 to 2002 (APC = −0.2; 95% CI: −3.5 to 5.5, p = 0.91), followed by a steep drop to 6.3 in 2013 (APC = −6.3; 95% CI: −8.2 to −5.7, p < 0.01). There were no notable changes until 2021 (AAPC = 0.6; 95% CI: −3.7 to 4.0, p = 0.24), and it ultimately decreased to 6.1 in 2023 (APC = −8.5; 95% CI: −13.6 to −1.2, p = 0.002) (Figure 3, Supplemental Table 2 and 6).

#### Urbanization

The majority of fatalities that occurred between 1999 and 2020 were situated in urban areas (105,496), whereas the number of deaths in rural areas was recorded at 31,629.

In 1999, rural areas exhibited a higher AAMR than urban areas, with rates of 23.5 and 18.4, respectively. The AAMR for rural areas initially demonstrated no variation until 2004 (APC = 1.4; 95% CI: −0.9 to 5.1; p = 0.19), which was followed by a consistent decline to 10.4 in 2014 (APC = −5.9; 95% CI: −9.1 to −5.0; p < 0.01), with no significant fluctuations thereafter until 2020 (APC = 1.7; 95% CI: −0.9 to 8.4; p = 0.13).

**Figure 4.**
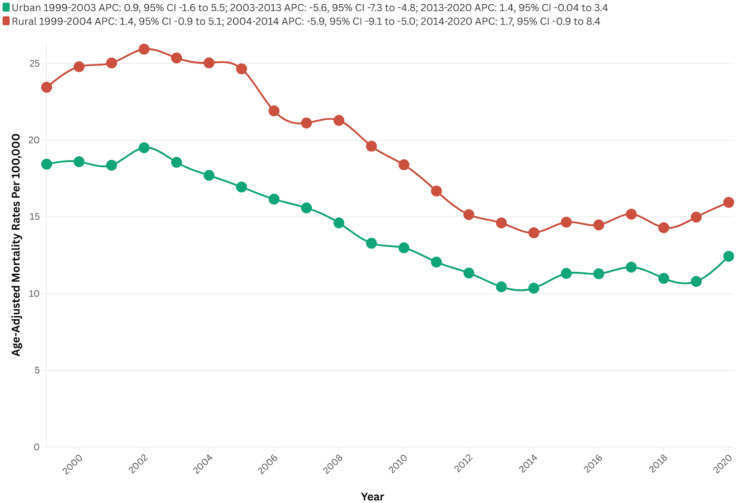
Urban-rural stratified AAMRs per 100,000 of stroke among old (65+) AD patients in the United States (1999–2023).

Likewise, the AAMR for urban areas exhibited no clinical significance until 2003 (APC = 0.9; 95% CI: −1.6 to 5.5; p = 0.45), followed by a substantial decrease to 14.6 in 2013 (APC = −5.3; 95% CI: −7.3 to −4.8, p < 0.01), ultimately in an increase to 16.0 in 2020 (APC = 1.4; 95% CI: −0.0 to 3.4; p = 0.05), as depicted in Figure 4.

Urban areas experienced a steeper decline over time (AAPC: −2.10, 95% CI: −2.5 to −1.6, p < 0.01) in comparison to rural areas (AAPC: −2.08, 95% CI: −2.6 to −1.5, p < 0.01) (Figure 4, Supplemental Table 2 and 7).

#### State

**Figure 5.**
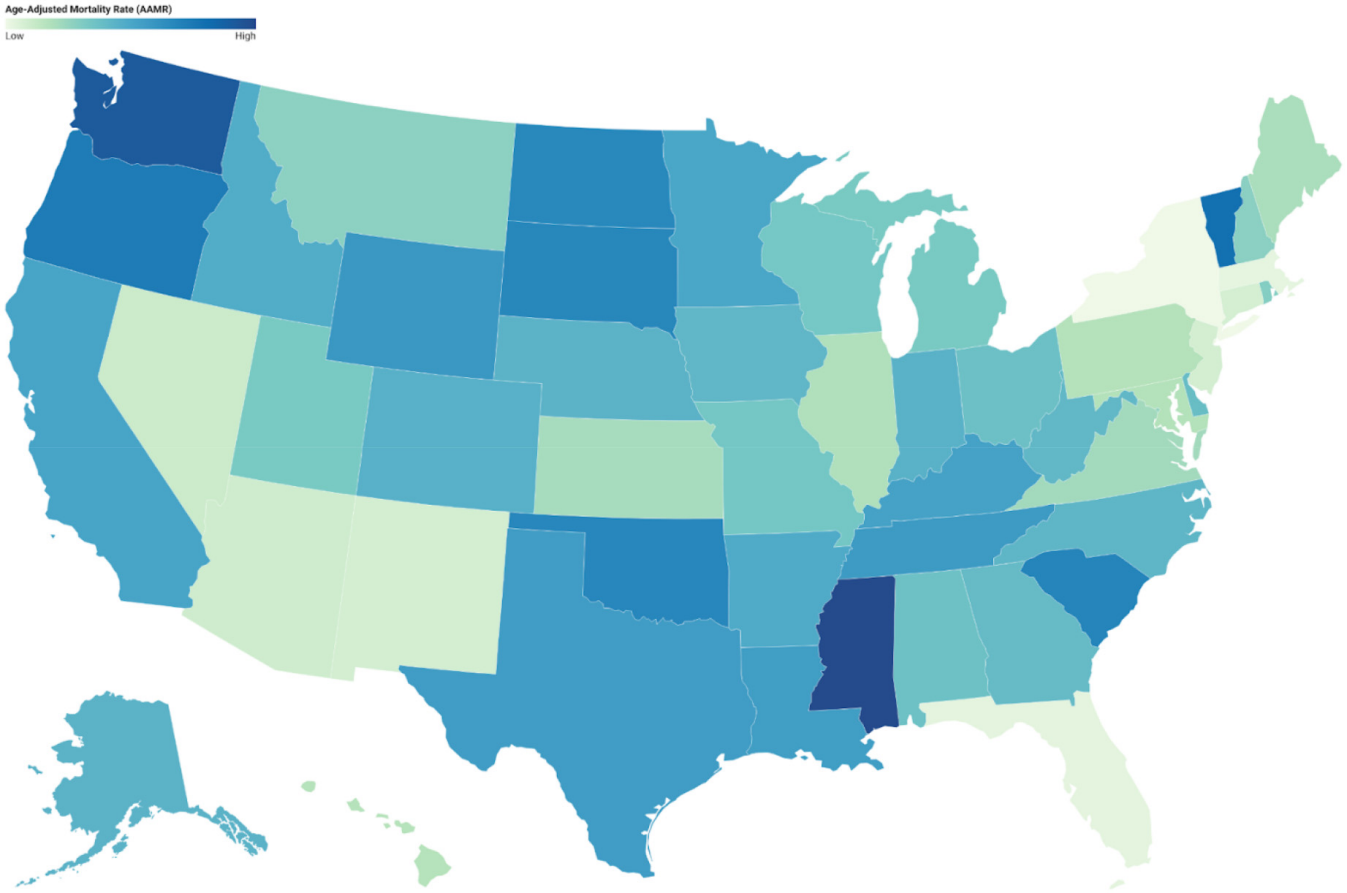
Age-adjusted mortality rates (AAMRs) per 100,000, by state, of stroke among old (65+) AD patients in the United States (1999–2023).

States within the top 90th percentile for mortality rates due to strokes and Alzheimer's disease include Mississippi (27.0), Washington (25.2), Vermont (22.7), Oregon (21.5), and South Carolina (20.4). In contrast, states within the lower 10th percentile comprise New Mexico (7.8), Connecticut (7.7), Florida (6.8), Massachusetts (6.7), and New York (6.1) (Figure 5, Supplemental Table 8).

## Discussion

**Figure 6.**
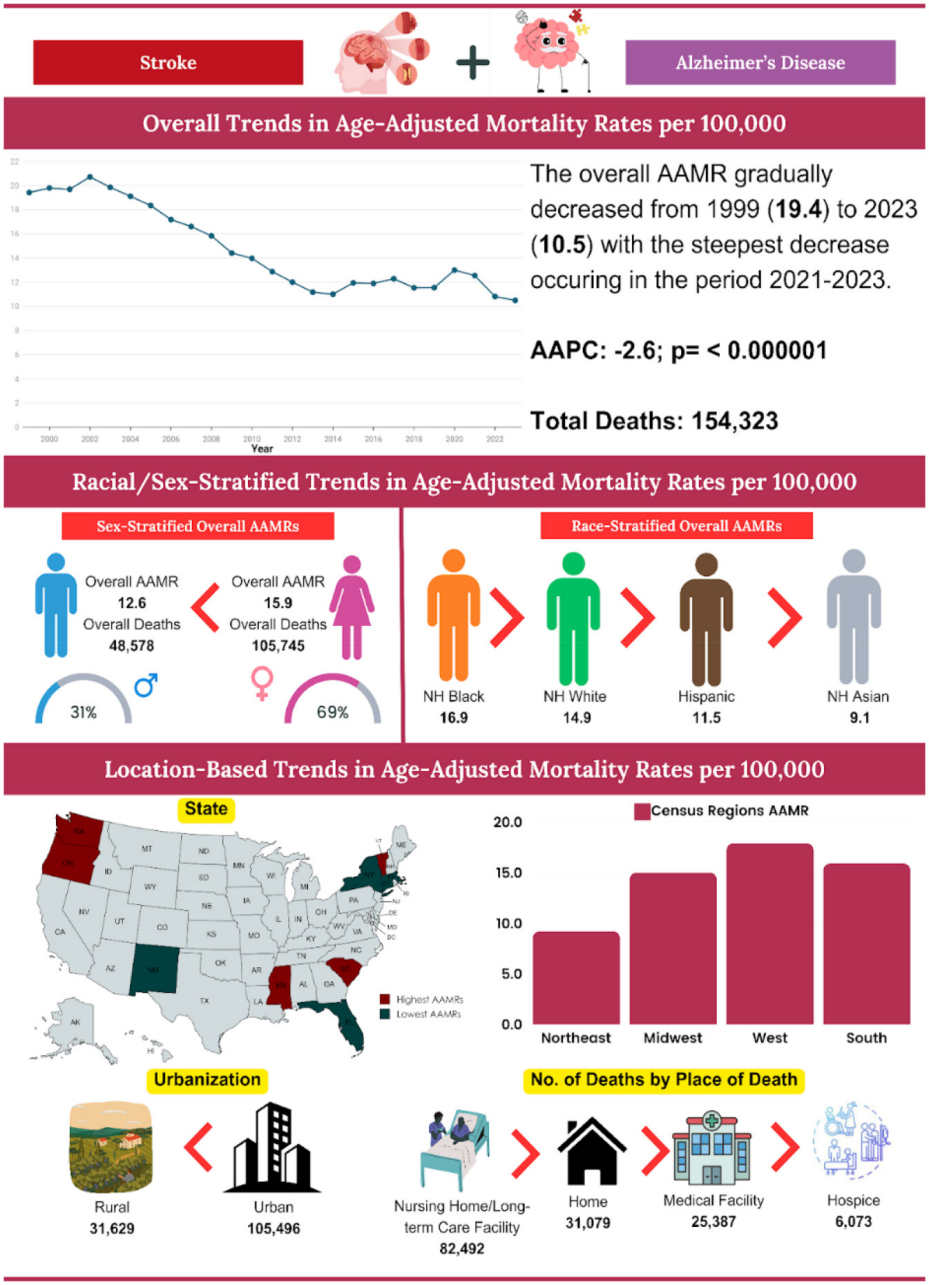
Central illustration.

The primary objective of this study was to investigate mortality trends among patients diagnosed with Alzheimer's disease who have also suffered a stroke, analyzing key demographic and geographic variables, including sex, race, place of death, and other relevant factors. Our analysis of data from the CDC between the years 1999 and 2023 revealed a total of 154,323 recorded deaths, with AAMRs demonstrating remarkable fluctuations. Initially, there was a slight increase in deaths from 1999 to 2003, followed by a sharp decline until 2013, then a period of stable resurgence from 2013 to 2021, and a subsequent steep decline thereafter from 2021 to 2023. Females represented a higher proportion of these deaths (105,745 compared to 48,578) and sustained elevated AAMRs (15.9 compared to 12.6) relative to males, although both genders experienced notable reductions in mortality over time. Racial disparities were evident, with NH Black individuals exhibiting the highest AAMR at 16.9, followed by NH Whites at 14.9, Hispanics at 11.5, and Asians/Pacific Islanders at 9.1. Geographically, the Southern region recorded the highest death count at 58,255, whereas the Western region exhibited the highest AAMR at 17.9. Urban areas accounted for the majority of absolute deaths, totaling 105,496; however, rural regions initially had higher AAMRs before aligning with urban trends. An analysis of the place of death indicated that nursing homes constituted the most frequent setting, representing 53.5% of cases (Figure 6: Central Illustration).

The decline in stroke-related mortality among AD patients between 1999 and 2023 elucidates the overall improvements in stroke prevention and management. Notably, the substantial decrease observed during the period from 1999 to 2013 can be attributed to advancements in stroke care, encompassing enhanced control of modifiable risk factors such as hypertension, diabetes, and dyslipidemia, alongside an increased utilization of thrombolysis (tPA) and endovascular thrombectomy.^20–22^ Moreover, the heightened awareness regarding stroke prevention within high-risk populations, including AD patients who frequently display cerebrovascular pathology, may have additionally contributed to this decline.^4,23^ Stroke is now recognized not only as a comorbidity but also as a risk factor for AD, owing to shared mechanisms such as cerebral hypoperfusion, neuro-inflammation, and amyloid angiopathy, which facilitate the acceleration of cognitive decline.^24–26^ The notable resurgence from 2013 to 2021 requires careful consideration, with one plausible explanation being the rising prevalence of AD associated with aging populations, compounded by improved diagnostic sensitivity through the widespread adoption of biomarker-based criteria.^27,28^ The adoption of more sensitive diagnostic criteria, such as the 2011 National Institute on Aging–Alzheimer's Association (NIA-AA) guidelines and the increasing use of neuroimaging and cerebrospinal fluid biomarkers, has led to earlier and more accurate identification of AD.^29,30^ These changes likely improved the documentation of comorbidities, including stroke, on death certificates. Furthermore, heightened awareness among clinicians and the integration of electronic medical records may have contributed to better reporting practices.^31^ As a result, what may appear as an actual rise in comorbid stroke-related mortality could, in part, be an artifact of evolving diagnostic and reporting standards.

Regarding the increase in 2021 itself, where the AAMR was recorded at 12.6, it reflects the impact of the COVID-19 pandemic.^32^ This period also witnessed an increase in obesity and metabolic syndrome rates, which may have counterbalanced previous advancements in managing vascular risks.^33^ Conversely, the subsequent significant decline observed from 2021 to 2023 may indicate disruptions caused by the COVID-19 pandemic, including underreporting of non-COVID deaths or alterations in healthcare accessibility^34,35^

Gender disparities correlate with significant sex-specific vulnerabilities associated with stroke and AD outcomes. The elevated mortality burden experienced by women is attributed to both biological and sociodemographic factors, including a longer life expectancy which exposes them to an extended risk of AD and stroke.^36–38^ Additionally, sex-specific cerebrovascular pathophysiology, such as the loss of estrogen post-menopause, exacerbates endothelial dysfunction.^39,40^ The disabilities that arise following a stroke are often more severe in women, due in part to age-related frailty and varied access to rehabilitation services, with a notable proportion of female stroke survivors experiencing vascular contributions to cognitive impairment.^41–43^ However, a study indicated that conjugated equine estrogens and medroxyprogesterone acetate may increase the risk of dementia in women aged 65 years and older, which is in contrast to earlier research that suggested estrogen therapy close to menopause might mitigate the risk of AD; this remains a subject of controversy.^44^ Epidemiological data indicate that women are disproportionately affected by mixed dementia, comprising both AD and vascular dementia, following ischemic injury.^45,46^

Racial disparities in stroke and AD mortality trends showed a significant public health concern, reflecting the enduring impact of racism, healthcare inequities, and social determinants of health. The highest AAMR of NH Black or African American individuals aligns with national data indicating Black Americans face a disproportionately higher risk of stroke, cognitive decline, and dementia-related death.^47–49^ Limited access to high-quality medical and psychological care and delayed diagnosis may cause this burden.^50–52^ Continuous exposure to racism and socioeconomic problems contributes to increased vascular risk factors, including hypertension, diabetes, and obesity, all of which are more prevalent among Black and Hispanic populations.^21,53^ Also, stress from racial discrimination can induce biological aging and cognitive problems.^54,55^ For Hispanic populations, while demonstrating lower overall AAMR, they have experienced some concerning temporal increases, possibly due to acculturation-related lifestyle changes.^56–58^ Asian Americans generally experienced lower AAMRs, though disparities persist in subgroups and access to culturally competent care.^46,59^ These results highlight the need for anti-racist public health policies, better representation in research, and focused preventive campaigns aiming at forward causes of stroke-related mortality and dementia in communities of color.

Geographical disparities in AD and stroke mortality showed the highest number of deaths in the South, but the West had the highest AAMR, followed by the South. These findings fit previous studies showing persistently higher rates of cerebrovascular disease and AD deaths in the Southern and Western.^60–62^ Lower socioeconomic conditions may cause the higher death rate in the South, no access to healthcare, and a higher incidence of vascular risk factors.^61,63,64^ Studies say that residents in the “Stroke Belt” of the Southeastern show significantly higher stroke mortality and earlier onset of cognitive impairment.^65–68^ The West's persistently high AAMR may reflect population aging, healthcare inequities among rural and Indigenous communities, and elevated AD diagnoses in states with high life expectancy.^69–72^ While the Northeast had the lowest AAMR, its sharp decline over time is consistent with regional strengths in healthcare quality, education, and public health funding.^68,72,73^ Prior research indicates that differences in Medicaid expansion, long-term care access, and AD awareness campaigns can explain these variations.^74,75^ Together, our findings underscore that regional context, including structural, behavioral, and policy-level determinants, plays an essential role in shaping mortality outcomes related to AD and stroke.

Urban-rural disparities in mortality rates from AD and stroke are increasingly acknowledged, as evidenced by systemic differences in healthcare access, socioeconomic conditions, and population aging. Although urban areas account for the majority of deaths, rural regions initially exhibited a higher AAMR in 1999. This early rural disadvantage aligns with previous findings, which indicate that rural populations consistently experience higher mortality from stroke and AD, attributed to limited access to specialists, a higher prevalence of comorbidities, and various socioeconomic challenges. Furthermore, over half of the cases of AD and ischemic stroke could potentially be averted if individuals possessed at least one protective factor and lacked any shared risk factors, thereby highlighting the significant influence of modifiable lifestyle and health factors on disease prevention.^76–79^ Over time, both regions saw AAMR declines, but urban areas experienced a slightly steeper decrease, aligning with literature noting greater healthcare infrastructure and dementia services in metropolitan centers.^80–82^ Rural communities continue to encounter significant barriers, including healthcare workforce shortages, fewer dementia-capable services, and lower health literacy.^83,84^ Conversely, urban areas, while better resourced, contend with higher population density and environmental stressors linked to neurodegeneration and some accidental problems.^85,86^

While the prevention of stroke in individuals with AD may intuitively seem beneficial—given its potential to reduce morbidity and preserve functional independence—it is essential to consider the broader ethical implications. AD is a progressive and ultimately terminal neurodegenerative condition, and in its advanced stages, the focus of care often shifts from prolonging life to prioritizing comfort and quality of life.^87^ Preventive strategies such as aggressive vascular risk factor management must therefore be guided by palliative principles, balancing potential benefits against treatment burdens, especially in frail or elderly patients.^88,89^ Families and caregivers commonly experience substantial emotional, financial, and physical strain, and interventions that extend life without preserving meaningful function may inadvertently intensify these burdens.^90^ A nuanced, patient-centered approach is thus critical—one that aligns stroke prevention and overall management with the expressed values, goals, and preferences of patients and their support networks.^91^

### Strengths and limitations

This study had many strengths. It used the CDC WONDER database, which is a comprehensive and reliable source of U.S. mortality data, covering 25 years (1999–2023). The use of AAMRs and Joinpoint analysis provides insights into long-term trends and disparities in stroke-related mortality among AD patients. The stratification of results by sex, race/ethnicity, geographic region, and urbanization highlights critical disparities, aligning with existing literature on the intersection of AD and stroke. Additionally, the large sample size (154,323 deaths) enhances the generalizability of the findings.

However, the study has some limitations. Since it relies on death certificate data, it may underreport whether AD or stroke is a contributing cause of mortality. As this study relies on mortality data based on ICD-10 coding from death certificates, some degree of misclassification bias is possible, particularly in distinguishing between types of dementia and stroke subtypes. Furthermore, the study lacks data on healthcare access, individual-level clinical data, such as comorbidities beyond those listed on death certificates, cognitive or functional status, socioeconomic status, or treatment history that could affect mortality trends. The underrepresentation of certain racial/ethnic groups (e.g., NH American Indians) limits causal inferences and comparisons with other racial groups. Patients who ultimately died of stroke may have experienced prior cerebrovascular events and that comorbid AD could have been under- or mis-documented in some instances. However, due to the use of de-identified, aggregate-level mortality data from the CDC WONDER database, we were limited to analyzing underlying cause-of-death coding rather than full clinical histories. Selection bias may be present due to the nature of mortality data, as stroke is typically recorded as a cause of death only in severe or fatal cases. This could lead to an overrepresentation of comorbid stroke in AD-related deaths, potentially skewing the observed trends and limiting the generalizability of the findings to all individuals with AD.

### Conclusion

This comprehensive analysis spanning 25 years reveals a notable reduction in stroke-related mortality among patients with AD; however, significant disparities persist across various factors, including sex, race, geography, and urbanization. Specifically, women, non-Hispanic Black individuals, and rural populations continue to be disproportionately affected, highlighting critical unmet public health needs. To effectively address the systemic disparities in stroke prevention and AD care, the findings advocate for targeted interventions. Future studies should incorporate clinical and socioeconomic data to enhance risk categorization and evaluate the impact of healthcare policy on these patterns. To reverse these trends and improve outcomes for high-risk groups, it is essential to implement equitable healthcare policies and tailored public health interventions.

## Supplemental Material

sj-docx-1-alz-10.1177_13872877251380689 - Supplemental material for Aging, Alzheimer's disease, and stroke: A 25-year longitudinal analysis of U.S. mortality trends Supplemental material, sj-docx-1-alz-10.1177_13872877251380689 for Aging, Alzheimer's disease, and stroke: A 25-year longitudinal analysis of U.S. mortality trends by Muhammad Faizan Ali, Husnain Ahmad, Mohamed Fawzi Hemida, Muhammad Faizan Tahir, Talha Qadeer, Sana Rasheed, Zarwa Rashid, Mohammad Hamza Bin Abdul Malik, Ashraf Ahmed, Alexander Morden, Muhammad Abdullah Naveed, Himaja Dutt Chigurupati and Sivaram Neppala in Journal of Alzheimer's Disease
