# Supplementary material for: Aging, Alzheimer's disease, and stroke: A 25-year longitudinal analysis of U.S. mortality trends
Source: J Alzheimers Dis. 2025 Sep 24;108(2):616–28. doi: 10.1177/13872877251380689 (PMC13187842; doi:10.1177/13872877251380689)
Supplement: sj-docx-1-alz-10.1177_13872877251380689 - Supplemental material for Aging, Alzheimer's disease, and stroke: A 25-year longitudinal analysis of U.S. mortality trends [file sj-docx-1-alz-10.1177_13872877251380689.docx]

**Supplemental Material**

**Aging, Alzheimer’s disease, and stroke: A 25-year longitudinal analysis of U.S. mortality trends**

**Supplemental Table 1.** Number of stroke among old (65+) Alzheimer’s adults-related deaths, stratified by sex and race in the United States (1999-2023)

| **Year** | **Overall** | **Women** | **Men** | **NH Whites** | **NH Blacks or African Americans** | **NH Asians or Pacific Islanders** | **NH American Indians or Alaska Natives** | **Hispanics or Latinos** | **Population** |
| --- | --- | --- | --- | --- | --- | --- | --- | --- | --- |
| **1999** | 6636 | 4585 | 2051 | 5895 | 500 | 58 | - | 158 | 34797841 |
| **2000** | 6870 | 4818 | 2052 | 6147 | 488 | 57 | 10 | 155 | 34991753 |
| **2001** | 6937 | 4929 | 2008 | 6131 | 541 | 58 | 14 | 172 | 35290291 |
| **2002** | 7399 | 5187 | 2212 | 6521 | 585 | 78 | 14 | 192 | 35522207 |
| **2003** | 7209 | 5033 | 2176 | 6279 | 608 | 71 | 19 | 222 | 35863529 |
| **2004** | 7026 | 4897 | 2129 | 6138 | 547 | 86 | 13 | 234 | 36203319 |
| **2005** | 6912 | 4868 | 2044 | 6005 | 567 | 89 | 16 | 230 | 36649798 |
| **2006** | 6623 | 4584 | 2039 | 5656 | 573 | 111 | 13 | 260 | 37164107 |
| **2007** | 6533 | 4555 | 1978 | 5581 | 590 | 101 | 12 | 245 | 37825711 |
| **2008** | 6359 | 4380 | 1979 | 5357 | 592 | 109 | 16 | 277 | 38777621 |
| **2009** | 5922 | 4151 | 1771 | 4983 | 548 | 109 | 16 | 256 | 39623175 |
| **2010** | 5838 | 3944 | 1894 | 4905 | 497 | 130 | 23 | 272 | 40267984 |
| **2011** | 5538 | 3841 | 1697 | 4610 | 496 | 139 | 13 | 278 | 41394141 |
| **2012** | 5308 | 3628 | 1680 | 4367 | 480 | 148 | 13 | 288 | 43145356 |
| **2013** | 5074 | 3472 | 1602 | 4172 | 464 | 133 | 12 | 284 | 44704074 |
| **2014** | 5063 | 3457 | 1606 | 4148 | 446 | 129 | 20 | 316 | 46243211 |
| **2015** | 5624 | 3816 | 1808 | 4550 | 523 | 157 | 18 | 364 | 47760852 |
| **2016** | 5711 | 3915 | 1796 | 4647 | 516 | 148 | 28 | 366 | 49244195 |
| **2017** | 6050 | 4064 | 1986 | 4865 | 592 | 169 | 17 | 398 | 50858679 |
| **2018** | 5824 | 3894 | 1930 | 4697 | 509 | 185 | 24 | 404 | 52431193 |
| **2019** | 5890 | 3887 | 2003 | 4690 | 582 | 179 | 21 | 418 | 54058263 |
| **2020** | 6779 | 4544 | 2235 | 5264 | 740 | 238 | 22 | 508 | 55659365 |
| **2021** | 6089 | 3994 | 2095 | 4687 | 665 | 213 | 24 | 461 | 55847953 |
| **2022** | 5667 | 3730 | 1937 | 4303 | 595 | 209 | 15 | 514 | 57794852 |
| **2023** | 5442 | 3572 | 1870 | 4232 | 526 | 207 | 24 | 423 | 59248361 |
| **Total** | **154323** | **105745** | **48578** | **128830** | **13770** | **3311** | **417** | **7695** | **1101367831** |

**Supplemental Table 2.** Annual percent change (APC) and average annual percent change (AAPC) of stroke among old (65+) Alzheimer’s adults-related age-adjusted mortality rates per 100,000 in the United States (1999-2023)

| **Year Interval** | **APC (95% CI)** | **Year Interval** | **AAPC (95% CI)** |
| --- | --- | --- | --- |
| **Overall** |  |  |  |
| 1999-2003 | 1.34 (-0.96 to 5.13) | 1999-2023 | -2.63* (-3.03 to -2.25) |
| 2003-2013 | -5.68* (-6.89 to -4.97) |  |  |
| 2013-2021 | 1.03* (0.13 to 3.49) |  |  |
| 2021-2023 | -9.06* (-13.62 to -3.51) |  |  |
| **Men** |  |  |  |
| 1999-2003 | 0.64 (-1.99 to 5.68) | 1999-2023 | -2.75* (-3.18 to -2.30) |
| 2003-2013 | -5.72* (-7.43 to -4.97) |  |  |
| 2013-2021 | 1.12* (0.09 to 4.07) |  |  |
| 2021-2023 | -9.22* (-14.08 to -3.19) |  |  |
| **Women** |  |  |  |
| 1999-2003 | 1.86 (-0.47 to 5.50) | 1999-2023 | -2.48* (-2.84 to -2.12) |
| 2003-2013 | -5.58* (-6.63 to -4.88) |  |  |
| 2013-2021 | 1.10* (0.17 to 3.09) |  |  |
| 2021-2023 | -9.05* (-12.98 to -3.32) |  |  |
| **NH Whites** |  |  |  |
| 1999-2003 | 0.98 (-1.38 to 5.00) | 1999-2023 | -2.72* (-3.19 to -2.30) |
| 2003-2013 | -5.83* (-7.31 to -5.07) |  |  |
| 2013-2021 | 1.08* (0.02 to 4.20) |  |  |
| 2021-2023 | -8.86* (-14.25 to -2.53) |  |  |
| **NH Asians or Pacific Islanders** |  |  |  |
| 1999-2023 | -1.18* (-1.76 to -0.45) | 1999-2023 | -1.18* (-1.76 to -0.45) |
| **NH Blacks or African Americans** |  |  |  |
| 1999-2006 | 1.24 (-1.07 to 4.94) | 1999-2023 | -2.17* (-2.83 to -1.50) |
| 2006-2014 | -6.57* (-12.04 to -4.63) |  |  |
| 2014-2021 | 3.34* (1.29 to 9.82) |  |  |
| 2021-2023 | -13.86* (-20.96 to -4.50) |  |  |
| **Hispanics or Latinos** |  |  |  |
| 1999-2004 | 4.61 (-0.14 to 15.62) | 1999-2023 | -0.59 (-1.20 to 0.40) |
| 2004-2013 | -3.81 (-10.67 to 0.02) |  |  |
| 2013-2021 | 1.59 (-4.40 to 7.83) |  |  |
| 2021-2023 | -6.99 (-13.47 to 0.41) |  |  |
| **Census Region 1 – Northeast** |  |  |  |
| 1999-2002 | -0.15 (-3.53 to 5.53) | 1999-2023 | -3.49* (-3.94 to -3.03) |
| 2002-2013 | -6.32* (-8.21 to -5.69) |  |  |
| 2013-2021 | 0.61 (-3.73 to 3.98) |  |  |
| 2021-2023 | -8.50* (-13.59 to -1.20) |  |  |
| **Census Region 2 – Midwest** |  |  |  |
| 1999-2002 | 2.05 (-2.21 to 9.48) | 1999-2023 | -2.62* (-3.17 to -2.14) |
| 2002-2014 | -5.42* (-7.03 to -4.82) |  |  |
| 2014-2020 | 3.15* (1.14 to 8.73) |  |  |
| 2020-2023 | -6.92* (-13.34 to -2.85) |  |  |
| **Census Region 3 – South** |  |  |  |
| 1999-2002 | 3.42 (-0.42 to 10.32) | 1999-2023 | -2.77* (-3.22 to -2.34) |
| 2002-2007 | -3.85* (-7.90 to -1.87) |  |  |
| 2007-2013 | -7.82 (-12.21 to 0.36) |  |  |
| 2013-2021 | 1.96* (0.91 to 4.84) |  |  |
| 2021-2023 | -11.60* (-16.65 to -5.60) |  |  |
| **Census Region 4 – West** |  |  |  |
| 1999-2002 | 1.97 (-0.35 to 5.89) | 1999-2023 | -2.10* (-2.44 to -1.73) |
| 2002-2014 | -4.11* (-5.17 to -3.50) |  |  |
| 2014-2020 | 0.29 (-0.91 to 3.23) |  |  |
| 2020-2023 | -7.02* (-10.91 to -2.09) |  |  |
| **Urban** |  |  |  |
| 1999-2003 | 0.89 (-1.62 to 5.54) | 1999-2020 | -2.10* (-2.46 to -1.65) |
| 2003-2013 | -5.62* (-7.30 to -4.85) |  |  |
| 2013-2020 | 1.38 (-0.04 to 3.39) |  |  |
| **Rural** |  |  |  |
| 1999-2004 | 1.35 (-0.93 to 5.08) | 1999-2020 | -2.08* (-2.56 to -1.55) |
| 2004-2014 | -5.92* (-9.10 to -4.98) |  |  |
| 2014-2020 | 1.72 (-0.86 to 8.42) |  |  |

*statistically significant value (p < 0.05)

**Supplemental Table 3.** Stroke among old (65+) Alzheimer’s adults-related mortality, stratified by place of death in the United States (1999-2023)

|  | **Deaths** | | | | | |
| --- | --- | --- | --- | --- | --- | --- |
| **Year** | **Medical Facility** | **Nursing Home/Long-term Care Facility** | **Hospices** | **Home** | **Other/Unknown** |  |
| **1999** | 1531 | 4184 | - | 716 | 203 |  |
| **2000** | 1520 | 4251 | - | 851 | 239 |  |
| **2001** | 1589 | 4253 | - | 841 | 249 |  |
| **2002** | 1563 | 4541 | - | 966 | 327 |  |
| **2003** | 1535 | 4397 | 13 | 920 | 344 |  |
| **2004** | 1405 | 4136 | 36 | 1073 | 369 |  |
| **2005** | 1341 | 4109 | 84 | 1040 | 338 |  |
| **2006** | 1268 | 3828 | 108 | 1063 | 356 |  |
| **2007** | 1282 | 3703 | 154 | 1072 | 322 |  |
| **2008** | 1120 | 3581 | 172 | 1098 | 388 |  |
| **2009** | 1000 | 3204 | 179 | 1075 | 464 |  |
| **2010** | 986 | 3200 | 207 | 1107 | 337 |  |
| **2011** | 833 | 3052 | 254 | 1073 | 326 |  |
| **2012** | 801 | 2891 | 214 | 1089 | 311 |  |
| **2013** | 737 | 2622 | 282 | 1091 | 341 |  |
| **2014** | 661 | 2577 | 306 | 1188 | 323 |  |
| **2015** | 661 | 2806 | 419 | 1346 | 387 |  |
| **2016** | 673 | 2772 | 476 | 1391 | 391 |  |
| **2017** | 695 | 2932 | 429 | 1532 | 457 |  |
| **2018** | 675 | 2795 | 437 | 1507 | 405 |  |
| **2019** | 659 | 2836 | 432 | 1558 | 402 |  |
| **2020** | 748 | 2989 | 452 | 2063 | 521 |  |
| **2021** | 750 | 2356 | 478 | 2030 | 471 |  |
| **2022** | 666 | 2196 | 469 | 1806 | 527 |  |
| **2023** | 688 | 2281 | 472 | 1583 | 414 |  |
| **Total** | **25387** | **82492** | **6073** | **31079** | **9212** |  |

**Supplemental Table 4.** Overall and sex‐stratified stroke among old (65+) Alzheimer’s adults-related age-adjusted mortality rates per 100,000 in the United States (1999-2023)

| **Age-Adjusted Rate (95% CI)** | | | |
| --- | --- | --- | --- |
| **Year** | **Men** | **Women** | **Overall** |
| **1999** | 17.32 (16.56 to 18.08) | 20.23 (19.64 to 20.82) | 19.42 (18.96 to 19.89) |
| **2000** | 17.16 (16.41 to 17.91) | 20.99 (20.4 to 21.59) | 19.8 (19.33 to 20.27) |
| **2001** | 16.33 (15.61 to 17.06) | 21.18 (20.59 to 21.78) | 19.69 (19.22 to 20.15) |
| **2002** | 17.86 (17.11 to 18.62) | 22.1 (21.5 to 22.7) | 20.72 (20.25 to 21.19) |
| **2003** | 17.06 (16.33 to 17.78) | 21.23 (20.64 to 21.82) | 19.86 (19.4 to 20.32) |
| **2004** | 16.37 (15.67 to 17.08) | 20.47 (19.9 to 21.05) | 19.11 (18.67 to 19.56) |
| **2005** | 15.24 (14.57 to 15.9) | 19.9 (19.34 to 20.46) | 18.35 (17.92 to 18.78) |
| **2006** | 14.86 (14.22 to 15.51) | 18.35 (17.82 to 18.89) | 17.19 (16.77 to 17.6) |
| **2007** | 13.95 (13.33 to 14.57) | 17.94 (17.42 to 18.47) | 16.61 (16.21 to 17.01) |
| **2008** | 13.65 (13.05 to 14.25) | 16.98 (16.47 to 17.49) | 15.84 (15.45 to 16.23) |
| **2009** | 11.87 (11.31 to 12.42) | 15.77 (15.29 to 16.26) | 14.42 (14.05 to 14.79) |
| **2010** | 12.42 (11.86 to 12.98) | 14.74 (14.27 to 15.21) | 13.98 (13.62 to 14.34) |
| **2011** | 10.74 (10.23 to 11.25) | 14.1 (13.65 to 14.55) | 12.88 (12.54 to 13.22) |
| **2012** | 10.34 (9.85 to 10.84) | 12.99 (12.56 to 13.42) | 12.01 (11.69 to 12.34) |
| **2013** | 9.47 (9 to 9.94) | 12.15 (11.74 to 12.56) | 11.18 (10.87 to 11.49) |
| **2014** | 9.27 (8.82 to 9.73) | 11.94 (11.54 to 12.35) | 11.01 (10.7 to 11.31) |
| **2015** | 10.13 (9.66 to 10.59) | 12.95 (12.53 to 13.36) | 11.95 (11.63 to 12.26) |
| **2016** | 9.84 (9.38 to 10.29) | 13.06 (12.64 to 13.47) | 11.9 (11.59 to 12.21) |
| **2017** | 10.5 (10.03 to 10.96) | 13.36 (12.94 to 13.78) | 12.29 (11.97 to 12.6) |
| **2018** | 9.88 (9.44 to 10.32) | 12.56 (12.16 to 12.96) | 11.54 (11.24 to 11.84) |
| **2019** | 10.01 (9.57 to 10.45) | 12.47 (12.08 to 12.87) | 11.55 (11.26 to 11.85) |
| **2020** | 10.83 (10.38 to 11.28) | 14.38 (13.96 to 14.81) | 13.01 (12.7 to 13.32) |
| **2021** | 10.76 (10.3 to 11.23) | 13.63 (13.21 to 14.06) | 12.55 (12.24 to 12.87) |
| **2022** | 9.37 (8.95 to 9.79) | 11.64 (11.26 to 12.01) | 10.82 (10.54 to 11.1) |
| **2023** | 8.85 (8.45 to 9.26) | 11.52 (11.14 to 11.9) | 10.5 (10.22 to 10.78) |
| **Total** | **12.56 (12.00 to 13.12)** | **15.87 (15.39 to 16.35)** | **14.73 (14.36 to 15.09)** |

**Supplemental Table 5.** Stroke among old (65+) Alzheimer’s adults-related age-adjusted mortality rates per 100,000, stratified by race in the United States (1999-2023)

| **Year** | **NH Whites** | **NH Blacks or African Americans** | **Hispanics or Latinos** | **NH Asians or Pacific Islanders** |
| --- | --- | --- | --- | --- |
| **1999** | 19.89 (19.38 to 20.4) | 19.45 (17.75 to 21.16) | 12.14 (10.22 to 14.05) | 9.84 (7.43 to 12.78) |
| **2000** | 20.49 (19.97 to 21) | 18.8 (17.13 to 20.47) | 11.56 (9.72 to 13.39) | 9.28 (7.01 to 12.05) |
| **2001** | 20.16 (19.65 to 20.66) | 20.71 (18.96 to 22.46) | 11.66 (9.9 to 13.43) | 8.64 (6.54 to 11.19) |
| **2002** | 21.23 (20.71 to 21.75) | 22.42 (20.6 to 24.24) | 12.7 (10.88 to 14.52) | 10.25 (8.07 to 12.83) |
| **2003** | 20.17 (19.67 to 20.67) | 22.92 (21.09 to 24.74) | 13.92 (12.07 to 15.77) | 9.21 (7.16 to 11.65) |
| **2004** | 19.48 (19 to 19.97) | 20.37 (18.66 to 22.08) | 14.06 (12.24 to 15.89) | 10.14 (8.09 to 12.56) |
| **2005** | 18.75 (18.28 to 19.23) | 20.74 (19.03 to 22.46) | 13 (11.31 to 14.7) | 9.91 (7.95 to 12.22) |
| **2006** | 17.25 (16.8 to 17.7) | 20.45 (18.77 to 22.13) | 13.71 (12.02 to 15.39) | 11.59 (9.42 to 13.76) |
| **2007** | 16.72 (16.28 to 17.16) | 20.56 (18.9 to 22.23) | 12.28 (10.73 to 13.83) | 9.73 (7.82 to 11.64) |
| **2008** | 15.8 (15.38 to 16.23) | 20.08 (18.46 to 21.7) | 13.14 (11.58 to 14.69) | 9.92 (8.05 to 11.79) |
| **2009** | 14.45 (14.05 to 14.86) | 18.08 (16.57 to 19.6) | 11.52 (10.11 to 12.94) | 9.26 (7.52 to 11.01) |
| **2010** | 14.05 (13.65 to 14.45) | 15.95 (14.54 to 17.36) | 11.58 (10.2 to 12.97) | 10.7 (8.86 to 12.54) |
| **2011** | 12.88 (12.5 to 13.25) | 15.39 (14.03 to 16.74) | 10.88 (9.6 to 12.16) | 10.32 (8.6 to 12.03) |
| **2012** | 11.99 (11.63 to 12.35) | 14.29 (13 to 15.57) | 10.6 (9.38 to 11.83) | 10.16 (8.52 to 11.8) |
| **2013** | 11.2 (10.85 to 11.54) | 13.3 (12.09 to 14.52) | 9.76 (8.62 to 10.9) | 8.31 (6.89 to 9.73) |
| **2014** | 11.07 (10.73 to 11.42) | 12.43 (11.27 to 13.59) | 10.03 (8.92 to 11.14) | 7.59 (6.28 to 8.9) |
| **2015** | 11.96 (11.61 to 12.31) | 14.1 (12.89 to 15.32) | 10.87 (9.75 to 11.99) | 8.43 (7.11 to 9.76) |
| **2016** | 12.03 (11.68 to 12.38) | 13.46 (12.29 to 14.63) | 10.37 (9.3 to 11.43) | 7.47 (6.26 to 8.67) |
| **2017** | 12.46 (12.11 to 12.81) | 14.97 (13.75 to 16.18) | 10.56 (9.52 to 11.61) | 7.94 (6.74 to 9.15) |
| **2018** | 11.78 (11.44 to 12.12) | 12.32 (11.24 to 13.4) | 10.25 (9.25 to 11.26) | 8.15 (6.97 to 9.33) |
| **2019** | 11.66 (11.33 to 12) | 13.64 (12.52 to 14.75) | 10.33 (9.34 to 11.33) | 7.49 (6.39 to 8.59) |
| **2020** | 12.93 (12.57 to 13.28) | 16.86 (15.64 to 18.08) | 11.89 (10.85 to 12.93) | 9.36 (8.17 to 10.55) |
| **2021** | 12.57 (12.21 to 12.93) | 15.99 (14.76 to 17.22) | 11.07 (10.06 to 12.09) | 9 (7.79 to 10.21) |
| **2022** | 10.69 (10.37 to 11.01) | 13.57 (12.47 to 14.67) | 11.49 (10.49 to 12.49) | 7.83 (6.76 to 8.89) |
| **2023** | 10.73 (10.41 to 11.05) | 11.87 (10.84 to 12.89) | 9.36 (8.46 to 10.25) | 7.67 (6.63 to 8.72) |
| **Total** | **14.90 (14.49 to 15.30)** | **16.91 (15.49 to 18.33)** | **11.55 (10.18 to 12.92)** | **9.13 97.48 to 10.89)** |

**Supplemental Table 6.** Stroke among old (65+) Alzheimer’s adults-related age-adjusted mortality rates per 100,000 stratified by census regions in the United States (1999-2023)

| **Age-Adjusted Rate (95% CI)** | | | | |
| --- | --- | --- | --- | --- |
| **Year** | **Northeast** | **Midwest** | **South** | **West** |
| **1999** | 13.9 (13.05 to 14.75) | 20.8 (19.82 to 21.77) | 20.49 (19.67 to 21.31) | 22.05 (20.91 to 23.18) |
| **2000** | 14.19 (13.34 to 15.04) | 20.05 (19.1 to 21) | 21.76 (20.92 to 22.6) | 22.32 (21.19 to 23.45) |
| **2001** | 13.66 (12.83 to 14.48) | 20.58 (19.62 to 21.53) | 21.86 (21.02 to 22.69) | 21.43 (20.34 to 22.52) |
| **2002** | 14.02 (13.19 to 14.85) | 21.55 (20.58 to 22.53) | 22.79 (21.94 to 23.64) | 23.48 (22.35 to 24.62) |
| **2003** | 12.84 (12.06 to 13.63) | 19.83 (18.9 to 20.76) | 22.32 (21.48 to 23.15) | 23.27 (22.16 to 24.39) |
| **2004** | 12.76 (11.97 to 13.54) | 19.04 (18.13 to 19.94) | 20.93 (20.13 to 21.73) | 22.74 (21.66 to 23.83) |
| **2005** | 11.25 (10.52 to 11.98) | 19.47 (18.56 to 20.38) | 20.41 (19.63 to 21.19) | 21.32 (20.28 to 22.35) |
| **2006** | 10.46 (9.77 to 11.16) | 17.72 (16.86 to 18.58) | 19.24 (18.49 to 19.99) | 20.21 (19.21 to 21.2) |
| **2007** | 9.9 (9.22 to 10.57) | 15.88 (15.08 to 16.69) | 19.17 (18.43 to 19.91) | 19.98 (19.01 to 20.96) |
| **2008** | 9.4 (8.75 to 10.05) | 15.77 (14.97 to 16.57) | 17.5 (16.8 to 18.2) | 19.67 (18.71 to 20.62) |
| **2009** | 8.75 (8.13 to 9.37) | 14.08 (13.33 to 14.84) | 15.46 (14.81 to 16.11) | 18.75 (17.83 to 19.67) |
| **2010** | 8.35 (7.74 to 8.96) | 13.64 (12.9 to 14.37) | 15.17 (14.53 to 15.81) | 17.95 (17.06 to 18.83) |
| **2011** | 8.06 (7.47 to 8.66) | 13.12 (12.4 to 13.83) | 13.22 (12.64 to 13.81) | 16.62 (15.77 to 17.46) |
| **2012** | 7.21 (6.66 to 7.76) | 12.5 (11.8 to 13.19) | 12.08 (11.53 to 12.63) | 15.98 (15.16 to 16.79) |
| **2013** | 6.32 (5.81 to 6.84) | 11.38 (10.73 to 12.03) | 11.56 (11.03 to 12.09) | 14.84 (14.07 to 15.61) |
| **2014** | 6.95 (6.41 to 7.5) | 10.87 (10.24 to 11.51) | 11.43 (10.91 to 11.95) | 14.08 (13.34 to 14.82) |
| **2015** | 7.43 (6.88 to 7.99) | 11.5 (10.85 to 12.15) | 12.42 (11.88 to 12.95) | 15.59 (14.82 to 16.36) |
| **2016** | 6.76 (6.23 to 7.29) | 11.69 (11.04 to 12.34) | 12.66 (12.12 to 13.19) | 15.26 (14.51 to 16.01) |
| **2017** | 7.04 (6.51 to 7.56) | 12.93 (12.26 to 13.61) | 12.98 (12.45 to 13.52) | 15.31 (14.56 to 16.05) |
| **2018** | 6.51 (6.01 to 7.02) | 11.53 (10.89 to 12.16) | 12.54 (12.03 to 13.06) | 14.64 (13.92 to 15.35) |
| **2019** | 6.68 (6.17 to 7.19) | 12.5 (11.84 to 13.16) | 11.95 (11.45 to 12.45) | 14.07 (13.38 to 14.77) |
| **2020** | 7.45 (6.91 to 7.98) | 13.68 (12.99 to 14.37) | 13.99 (13.46 to 14.52) | 15.57 (14.84 to 16.29) |
| **2021** | 7.34 (6.79 to 7.89) | 12.89 (12.2 to 13.58) | 13.33 (12.8 to 13.87) | 15.35 (14.6 to 16.09) |
| **2022** | 6.19 (5.71 to 6.68) | 10.69 (10.08 to 11.3) | 11.46 (10.99 to 11.94) | 13.75 (13.08 to 14.42) |
| **2023** | 6.12 (5.63 to 6.61) | 11.06 (10.44 to 11.69) | 10.75 (10.29 to 11.21) | 13.22 (12.56 to 13.89) |
| **Total** | **9.18 (8.55 to 9.81)** | **14.99 (14.22 to 15.76)** | **15.90 (15.26 to 16.54)** | **17.90 (17.01 to 18.78)** |

**Supplemental Table 7.** Stroke among old (65+) Alzheimer’s adults-related age-adjusted mortality rates per 100,000 in the urban and rural areas in the United States (1999-2020)

|  | **Age-Adjusted Rate (95% CI)** | |
| --- | --- | --- |
| **Year** | **Urban** | **Rural** |
| **1999** | 18.44 (17.93 to 18.95) | 23.45 (22.3 to 24.61) |
| **2000** | 18.6 (18.1 to 19.11) | 24.79 (23.61 to 25.98) |
| **2001** | 18.37 (17.87 to 18.87) | 25.03 (23.84 to 26.21) |
| **2002** | 19.51 (19 to 20.02) | 25.93 (24.72 to 27.13) |
| **2003** | 18.56 (18.07 to 19.06) | 25.36 (24.17 to 26.55) |
| **2004** | 17.71 (17.23 to 18.18) | 25.04 (23.86 to 26.22) |
| **2005** | 16.95 (16.49 to 17.41) | 24.65 (23.48 to 25.81) |
| **2006** | 16.16 (15.71 to 16.6) | 21.91 (20.82 to 23) |
| **2007** | 15.59 (15.16 to 16.02) | 21.12 (20.06 to 22.18) |
| **2008** | 14.61 (14.2 to 15.03) | 21.29 (20.23 to 22.35) |
| **2009** | 13.29 (12.9 to 13.68) | 19.61 (18.6 to 20.63) |
| **2010** | 12.99 (12.61 to 13.37) | 18.4 (17.42 to 19.37) |
| **2011** | 12.06 (11.69 to 12.42) | 16.69 (15.76 to 17.61) |
| **2012** | 11.35 (11 to 11.69) | 15.15 (14.28 to 16.03) |
| **2013** | 10.45 (10.12 to 10.78) | 14.61 (13.77 to 15.46) |
| **2014** | 10.36 (10.04 to 10.69) | 13.97 (13.14 to 14.79) |
| **2015** | 11.32 (10.98 to 11.65) | 14.66 (13.82 to 15.5) |
| **2016** | 11.3 (10.97 to 11.64) | 14.48 (13.65 to 15.3) |
| **2017** | 11.73 (11.4 to 12.07) | 15.18 (14.33 to 16.02) |
| **2018** | 11 (10.68 to 11.32) | 14.3 (13.5 to 15.11) |
| **2019** | 10.8 (10.49 to 11.12) | 14.99 (14.17 to 15.81) |
| **2020** | 12.44 (12.11 to 12.77) | 15.95 (15.11 to 16.79) |
| **Total** | **14.25 (13.85 to 14.66)** | **19.39 (18.39 to 20.38)** |

**Supplemental Table 8.** Stroke among old (65+) Alzheimer’s adults-related age-adjusted mortality rates per 100,000, by state in the United States (1999-2023)

| **State** | **Rank** | **Deaths** | **Age Adjusted Rate (95% CI)** | **Percentile** |
| --- | --- | --- | --- | --- |
| **Mississippi** | 1 | 2581 | 27.01 (25.03 to 28.99) | 98 |
| **Washington** | 2 | 5853 | 25.225 (24.015 to 26.435) | 96 |
| **Vermont** | 3 | 591 | 22.73 (19.395 to 26.505) | 94 |
| **Oregon** | 4 | 2872 | 21.465 (20.01 to 22.92) | 92 |
| **South Carolina** | 5 | 3181 | 20.435 (19.14 to 21.735) | 90 |
| **Oklahoma** | 6 | 2698 | 20.225 (18.76 to 21.69) | 88 |
| **South Dakota** | 7 | 692 | 20.155 (17.32 to 23.345) | 86 |
| **North Dakota** | 8 | 680 | 20 (17.155 to 23.235) | 84 |
| **Wyoming** | 9 | 297 | 18.325 (14.71 to 22.535) | 82 |
| **Tennessee** | 10 | 4655 | 18.02 (17.04 to 19) | 80 |
| **Texas** | 11 | 12082 | 17.705 (17.12 to 18.285) | 78 |
| **Louisiana** | 12 | 2441 | 17.7 (16.375 to 19.02) | 76 |
| **Kentucky** | 13 | 2770 | 17.155 (15.925 to 18.385) | 75 |
| **California** | 14 | 22216 | 16.835 (16.415 to 17.255) | 73 |
| **Minnesota** | 15 | 3045 | 16.69 (15.585 to 17.79) | 71 |
| **Arkansas** | 16 | 1929 | 16.155 (14.765 to 17.55) | 69 |
| **Idaho** | 17 | 805 | 16.035 (14.03 to 18.05) | 67 |
| **Colorado** | 18 | 2117 | 15.555 (14.36 to 16.755) | 65 |
| **Indiana** | 19 | 3775 | 15.385 (14.44 to 16.34) | 63 |
| **Nebraska** | 20 | 1128 | 15.295 (13.535 to 17.055) | 61 |
| **Alaska** | 21 | 157 | 15.27 (12.72 to 17.81) | 59 |
| **North Carolina** | 22 | 5367 | 14.985 (14.23 to 15.735) | 57 |
| **Iowa** | 23 | 2181 | 14.87 (13.63 to 16.11) | 55 |
| **West Virginia** | 24 | 1326 | 14.73 (13.225 to 16.395) | 53 |
| **Georgia** | 25 | 4109 | 14.44 (13.625 to 15.255) | 51 |
| **Delaware** | 26 | 431 | 14.12 (11.82 to 16.72) | 50 |
| **Ohio** | 27 | 7276 | 13.81 (13.185 to 14.43) | 48 |
| **Alabama** | 28 | 2763 | 13.735 (12.765 to 14.705) | 46 |
| **Missouri** | 29 | 3418 | 13.175 (12.315 to 14.04) | 44 |
| **Wisconsin** | 30 | 2855 | 13.04 (12.115 to 13.97) | 42 |
| **Michigan** | 31 | 4825 | 12.805 (12.105 to 13.505) | 40 |
| **Utah** | 32 | 815 | 12.8 (11.19 to 14.405) | 38 |
| **Rhode Island** | 33 | 531 | 12.295 (10.36 to 14.49) | 36 |
| **New Hampshire** | 34 | 669 | 11.97 (10.33 to 13.83) | 34 |
| **Montana** | 35 | 537 | 11.885 (10.05 to 14) | 32 |
| **District of Columbia** | 36 | 202 | 11.46 (9.81 to 13.1) | 30 |
| **Virginia** | 37 | 2917 | 10.645 (9.93 to 11.36) | 28 |
| **Kansas** | 38 | 1275 | 10.395 (9.26 to 11.53) | 26 |
| **Maine** | 39 | 675 | 10.18 (8.77 to 11.775) | 25 |
| **Illinois** | 40 | 5330 | 9.93 (9.415 to 10.45) | 23 |
| **Maryland** | 41 | 2049 | 9.66 (8.88 to 10.45) | 21 |
| **Pennsylvania** | 42 | 6200 | 9.635 (9.16 to 10.115) | 19 |
| **Hawaii** | 43 | 605 | 9.55 (8.215 to 11.045) | 17 |
| **Nevada** | 44 | 513 | 8.295 (7.08 to 9.515) | 15 |
| **Arizona** | 45 | 2245 | 8.055 (7.45 to 8.66) | 13 |
| **New Jersey** | 46 | 3034 | 7.8 (7.26 to 8.34) | 11 |
| **New Mexico** | 47 | 585 | 7.795 (6.67 to 9.075) | 9 |
| **Connecticut** | 48 | 1496 | 7.73 (6.98 to 8.56) | 7 |
| **Florida** | 49 | 6754 | 6.8 (6.5 to 7.1) | 5 |
| **Massachusetts** | 50 | 2048 | 6.73 (6.155 to 7.305) | 3 |
| **New York** | 51 | 4727 | 6.06 (5.73 to 6.39) | 1 |

**Supplemental Table 9.** Sensitivity analysis of number of deaths and age-adjusted mortality rates for stroke subtypes among older (65+) adults with Alzheimer’s disease in the United States (1999–2023)

| **Year** | **Number of deaths** | | | **AAMR (95% CI)** | | |
| --- | --- | --- | --- | --- | --- | --- |
|  | **Hemorrhagic Stroke** | **Ischemic Stroke** | **Other Strokes** | **Hemorrhagic Stroke** | **Ischemic Stroke** | **Other Strokes** |
| **1999** | **436** | **765** | **6665** | **1.28 (1.16 – 1.40)** | **2.25 (2.09 – 2.41)** | **19.48 (19.02 – 19.95)** |
| **2000** | **496** | **766** | **6902** | **1.41 (1.28 – 1.53)** | **2.22 (2.07 – 2.38)** | **19.90 (19.43 – 20.36)** |
| **2001** | **488** | **699** | **7118** | **1.40 (1.28 – 1.53)** | **1.98 (1.83 – 2.13)** | **20.17 (19.71 – 20.64)** |
| **2002** | **540** | **724** | **7724** | **1.52 (1.39 – 1.64)** | **2.06 (1.91 – 2.21)** | **21.66 (21.17 – 22.14)** |
| **2003** | **592** | **709** | **7620** | **1.64 (1.51 – 1.77)** | **1.94 (1.80 – 2.08)** | **21.00 (20.53 – 21.47)** |
| **2004** | **621** | **708** | **7529** | **1.68 (1.55 – 1.82)** | **1.95 (1.80 – 2.09)** | **20.45 (19.98 – 20.91)** |
| **2005** | **598** | **464** | **7515** | **1.61 (1.48 – 1.74)** | **1.20 (1.09 – 1.31)** | **19.93 (19.47 – 20.38)** |
| **2006** | **582** | **416** | **7083** | **1.49 (1.37 – 1.61)** | **1.08 (0.98 – 1.18)** | **18.40 (17.97 – 18.83)** |
| **2007** | **597** | **378** | **7048** | **1.53 (1.40 – 1.65)** | **0.95 (0.85 – 1.04)** | **17.90 (17.48 – 18.32)** |
| **2008** | **605** | **390** | **6850** | **1.51 (1.39 – 1.63)** | **0.98 (0.89 – 1.08)** | **17.02 (16.62 – 17.43)** |
| **2009** | **569** | **343** | **6427** | **1.38 (1.27 – 1.50)** | **0.86 (0.77 – 0.95)** | **15.64 (15.26 – 16.03)** |
| **2010** | **607** | **335** | **6440** | **1.49 (1.37 – 1.61)** | **0.80 (0.72 – 0.89)** | **15.38 (15.00 – 15.76)** |
| **2011** | **615** | **323** | **6090** | **1.47 (1.35 – 1.58)** | **0.78 (0.69 – 0.86)** | **14.08 (13.72 – 14.43)** |
| **2012** | **542** | **295** | **5821** | **1.25 (1.14 – 1.36)** | **0.69 (0.61 – 0.78)** | **13.18 (12.84 – 13.52)** |
| **2013** | **559** | **277** | **5637** | **1.30 (1.19 – 1.41)** | **0.61 (0.54 – 0.68)** | **12.39 (12.07 – 12.72)** |
| **2014** | **592** | **295** | **5760** | **1.32 (1.22 – 1.43)** | **0.65 (0.57 – 0.72)** | **12.48 (12.15 – 12.80)** |
| **2015** | **599** | **376** | **6425** | **1.29 (1.18 – 1.39)** | **0.79 (0.71 – 0.87)** | **13.58 (13.24 – 13.91)** |
| **2016** | **670** | **579** | **6510** | **1.42 (1.31 – 1.53)** | **1.18 (1.08 – 1.28)** | **13.47 (13.14 – 13.80)** |
| **2017** | **761** | **794** | **6687** | **1.55 (1.44 – 1.67)** | **1.60 (1.49 – 1.72)** | **13.63 (13.30 – 13.96)** |
| **2018** | **747** | **975** | **6417** | **1.49 (1.39 – 1.60)** | **1.96 (1.83 – 2.08)** | **12.74 (12.43 – 13.06)** |
| **2019** | **742** | **1001** | **6662** | **1.51 (1.41 – 1.62)** | **1.97 (1.85 – 2.09)** | **13.01 (12.69 – 13.32)** |
| **2020** | **737** | **1406** | **7751** | **1.43 (1.33 – 1.54)** | **2.71 (2.57 – 2.85)** | **14.84 (14.51 – 15.17)** |
| **2021** | **1332** | **1332** | **7030** | **2.74 (2.59 – 2.88)** | **2.74 (2.59 – 2.88)** | **14.46 (14.12 – 14.80)** |
| **2022** | **1349** | **1349** | **6802** | **2.54 (2.40 – 2.68)** | **2.54 (2.40 – 2.68)** | **12.96 (12.65 – 13.27)** |
| **2023** | **1467** | **1467** | **6484** | **2.83 (2.69 – 2.98)** | **2.83 (2.69 – 2.98)** | **12.51 (12.20 – 12.81)** |
| **Total/ Average** | **17443** | **17166** | **168997** | **1.60 (1.48 – 1.72)** | **1.57 (1.45 – 1.68)** | **16.01 (15.62 – 19.95)** |

**Supplemental Table 10.** Sensitivity analysis of annual percent change (APC) and average annual percent change (AAPC) for stroke subtypes among older (65+) adults with Alzheimer’s disease in the United States (1999–2023)

| **Cohort** | **Year Interval** | **APC (95% CI)** | **Year Interval** | **AAPC (95% CI)** |
| --- | --- | --- | --- | --- |
| **Hemorrhagic Stroke** | 1999–2018 | –0.4784 (–1.9989 to 0.7098) | 1999–2023 | 2.8309* (1.977 to 3.7376) |
|  | 2018–2023 | 16.4414* (9.8437 to 31.4059) |  |  |
| **Ischemic Stroke** | 1999–2013 | –9.7818* (–12.2945 to –7.867) | 1999–2023 | 0.2866 (–0.7217 to 1.1793) |
|  | 2013–2020 | 23.8751* (18.4044 to 42.3016) |  |  |
|  | 2020–2023 | 0.3706 (–12.4999 to 10.4427) |  |  |
| **Other Strokes** | 1999–2003 | 2.9733* (0.4905 to 7.3366) | 1999–2023 | –1.8562* (–2.2547 to –1.4372) |
|  | 2003–2013 | –5.2689* (–6.5617 to –4.4775) |  |  |
|  | 2013–2021 | 1.4410* (0.2641 to 4.2053) |  |  |
|  | 2021–2023 | –6.7624* (–11.3859 to –0.964) |  |  |

**STROBE Statement—checklist of items that should be included in reports of observational studies**

|  | Item No. | Recommendation | Page  No. | Relevant text from manuscript |
| --- | --- | --- | --- | --- |
| **Title and abstract** | 1 | (*a*) Indicate the study’s design with a commonly used term in the title or the abstract | 1,2 |  |
|  |  | (*b*) Provide in the abstract an informative and balanced summary of what was done and what was found | 2,3 |  |
| Introduction | | | |  |
| Background/rationale | 2 | Explain the scientific background and rationale for the investigation being reported | 4 |  |
| Objectives | 3 | State specific objectives, including any prespecified hypotheses | 4 |  |
| Methods | | | |  |
| Study design | 4 | Present key elements of study design early in the paper | 5 |  |
| Setting | 5 | Describe the setting, locations, and relevant dates, including periods of recruitment, exposure, follow-up, and data collection | 5 |  |
| Participants | 6 | a)      ***Cohort study***—Give the eligibility criteria, and the sources and methods of selection of participants. Describe methods of follow-up  b)      ***Case-control study***—Give the eligibility criteria, and the sources and methods of case ascertainment and control selection. Give the rationale for the choice of cases and controls  c)      ***Cross-sectional study***—Give the eligibility criteria, and the sources and methods of selection of participants | 5 |  |
|  |  | d)      ***Cohort study***—For matched studies, give matching criteria and number of exposed and unexposed  *e)*  ***Case-control study***—For matched studies, give matching criteria and the number of controls per case |  |  |
| Variables | 7 | Clearly define all outcomes, exposures, predictors, potential confounders, and effect modifiers. Give diagnostic criteria, if applicable | 6,7 |  |
| Data sources/ measurement | 8* | For each variable of interest, give sources of data and details of methods of assessment (measurement). Describe comparability of assessment methods if there is more than one group | *6* |  |
| Bias | 9 | Describe any efforts to address potential sources of bias | 6,7 |  |
| Study size | 10 | Explain how the study size was arrived at | 6,7 |  |

| Quantitative variables | | 11 | | Explain how quantitative variables were handled in the analyses. If applicable, describe which groupings were chosen and why | 6,7 | |  |
| --- | --- | --- | --- | --- | --- | --- | --- |
| Statistical methods | | 12 | | (*a*) Describe all statistical methods, including those used to control for confounding | 6 | |  |
|  |  |  |  | (*b*) Describe any methods used to examine subgroups and interactions | 6,7 | |  |
|  |  |  |  | (*c*) Explain how missing data were addressed | 6,7 | |  |
|  |  |  |  | (*d*) *Cohort study*—If applicable, explain how loss to follow-up was addressed  *Case-control study*—If applicable, explain how matching of cases and controls was addressed  *Cross-sectional study*—If applicable, describe analytical methods taking account of sampling strategy | 6 | |  |
|  |  |  |  | (*e*) Describe any sensitivity analyses | Not Applicable | |  |
| Results | | | | | | | |
| Participants | | 13* | | (a) Report numbers of individuals at each stage of study—eg numbers potentially eligible, examined for eligibility, confirmed eligible, included in the study, completing follow-up, and analysed | 8 | |  |
|  |  |  |  | (b) Give reasons for non-participation at each stage | Not Applicable | |  |
|  |  |  |  | (c) Consider use of a flow diagram | Not Applicable | |  |
| Descriptive data | | 14* | | (a) Give characteristics of study participants (eg demographic, clinical, social) and information on exposures and potential confounders | 8 | |  |
|  |  |  |  | (b) Indicate number of participants with missing data for each variable of interest | Nil | |  |
|  |  |  |  | (c) *Cohort study*—Summarise follow-up time (eg, average and total amount) | Not Applicable | |  |
| Outcome data | | 15* | | *Cohort study*—Report numbers of outcome events or summary measures over time | Not Applicable | |  |
|  |  |  |  | *Case-control study—*Report numbers in each exposure category, or summary measures of exposure | Not Applicable | |  |
|  |  |  |  | *Cross-sectional study—*Report numbers of outcome events or summary measures | *7,8* | |  |
| Main results | | 16 | | (*a*) Give unadjusted estimates and, if applicable, confounder-adjusted estimates and their precision (eg, 95% confidence interval). Make clear which confounders were adjusted for and why they were included | 8-15 | |  |
|  |  |  |  | (*b*) Report category boundaries when continuous variables were categorized | Not Applicable | |  |
|  |  |  |  | (*c*) If relevant, consider translating estimates of relative risk into absolute risk for a meaningful time period | Not Applicable | |  |
| Other analyses | 17 | | Report other analyses done—eg analyses of subgroups and interactions, and sensitivity analyses | | Nil |  | |
| Discussion | | | | | | | |
| Key results | 18 | | Summarise key results with reference to study objectives | | 15-17 |  | |
| Limitations | 19 | | Discuss limitations of the study, taking into account sources of potential bias or imprecision. Discuss both direction and magnitude of any potential bias | | 19 |  | |
| Interpretation | 20 | | Give a cautious overall interpretation of results considering objectives, limitations, multiplicity of analyses, results from similar studies, and other relevant evidence | | 15-20 |  | |
| Generalisability | 21 | | Discuss the generalisability (external validity) of the study results | | Not Applicable |  | |
| Other information | | |  | | | | |
| Funding | 22 | | Give the source of funding and the role of the funders for the present study and, if applicable, for the original study on which the present article is based | | none |  | |
|  |  |  |  |  |  |  |  |

***Give information separately for cases and controls in case-control studies and, if applicable, for exposed and unexposed groups in cohort and cross-sectional studies.**
